# Case report: Individualized treatment of advanced breast cancer with the use of the patient-derived tumor-like cell cluster model

**DOI:** 10.3389/fonc.2022.897984

**Published:** 2022-10-31

**Authors:** Wenjie Xia, Wuzhen Chen, Shan Fang, Jun Wu, Jingxia Zhang, Hongjun Yuan

**Affiliations:** ^1^ General Surgery, Cancer Center, Department of Breast Surgery, Zhejiang Provincial People’s Hospital, Affiliated People’s Hospital, Hangzhou Medical College, Hangzhou, China; ^2^ Department of Breast Surgery (Surgical Oncology), Second Affiliated Hospital, Zhejiang University School of Medicine, Hangzhou, China; ^3^ Rehabilitation Medicine Center, Rehabilitation and Sports Medicine Research Institute of Zhejiang Province, Department of Rehabilitation Medicine, Zhejiang Provincial People’s Hospital, Affiliated People’s Hospital, Hangzhou Medical College, Hangzhou, China

**Keywords:** breast cancer, individualized treatment, patient-derived tumor model, drug testing, case report

## Abstract

Breast cancer is one of the most common tumors in women. Despite various treatments, the survival of patients with advanced breast cancer is still disappointing. Furthermore, finding an effective individualized treatment for different kinds of patients is a thorny problem. Patient-derived tumor-like cell clusters were reported to be used for personalized drug testing in cancer therapy and had a prediction accuracy of 93%. However, there is still a lack of case reports about its application in the individualized treatment of breast cancer patients. Here, we described four cases of individualized treatment for advanced breast cancer using the patient-derived tumor-like cell cluster model (PTC model). In these four cases, the PTC model showed a good predictive effect. The tumor size was reduced significantly or even disappeared completely through clinical, radiological, or pathological evaluation with the help of the PTC model for selecting an individualized therapy regimen. Furthermore, the drug sensitivity test results of the PTC model were consistent with pathological molecular typing and the actual clinical drug resistance of the patients. In summary, our case report first evaluated the application value of the PTC model in advanced breast cancer, and the PTC model might be used as an efficient tool for drug resistance screening and for selecting a better personalized treatment, although further study is needed to prove the validity and stability of the PTC model in drug screening.

## Introduction

Breast cancer is one of the most common tumors, and its incidence rate ranks first in female malignant tumors (1). Despite various treatments, the survival of patients with advanced breast cancer is still disappointing, and the overall survival (OS) is approximately 31% (2). Because of the rapid progression of the tumor, it is of great importance to find an effective treatment in time (3). However, finding an effective individualized treatment for different kinds of patients is a thorny problem (4, 5). Some articles reported that patient-derived tumor-like cell clusters could be used for personalized drug testing in cancer therapy and had a prediction accuracy of 93% (6). However, there is still a lack of case reports about its application in the individualized treatment of breast cancer patients. Here, we described four cases of individualized treatment for advanced breast cancer with the use of the patient-derived tumor-like cell cluster model (PTC model).

## Methods

To identify the optimum therapy for individualized treatment, a personalized PTC drug testing system was conducted as described in a previous study (6). Thousands of PTCs were divided into a multiwell chip and were evaluated with different drugs, and it was confirmed that the gene expressions within different wells were highly correlated and PTC gene expressions were consistent with that of the original tumor (6). In clinical practice, the Response Evaluation Criteria in Solid Tumors (RECIST) is usually used to assess the efficacy of individualized treatment. The PTC model defined a similar method to assess drug efficacy: it first fixed the cutoff value of cell viability and then determined the effective concentrations of different drugs.

First, drug efficacy was assessed by measuring the area of all PTCs in each well. PTCs were photographed and evaluated on days 0 and 7. Only cell clusters with diameters greater than 40 μm when measured at both time points were used to estimate the total area. Moreover, cell viability after the addition of drug A was estimated by the following method:


$$p_{Ai}=S_{Ai,q1}/S_{Ai,q0};p_{A}=1/n\sum_{i=1}^{n}pAi$$

where *S* represents the sum of the cluster area in each well, *n* represents the number of repetitions, and q0, q1 represent the time points (days 0 and 7) when the area is measured. The cell viability of the negative control (pNC) was calculated in the same way and served as a quality control. If pNC was less than 0.9, the PTC test was discarded because PTCs were possibly in the decline phase.

Second, the cutoff value of the PTC model was determined based on the RECIST criteria as described in a previous study (6). According to the RECIST criteria, the tumor efficacy was divided into two subgroups with 0.7 as the cutoff value, and partial response (PR) or complete response (CR) was regarded as effective, while progressive disease (PD) or stable disease (SD) as otherwise. Accordingly, the drug was regarded as effective if pA<0.7, and the drug was not effective if pA ≥0.7.

Lastly, the effective drug concentration (Ec) of drug A in the PTC model was determined according to its clinical efficacy (6). The clinical efficacy was assessed in 272 breast cancer patients admitted to Zhejiang Provincial People’s Hospital from 1 January 2014 to 31 December 2020 with neoadjuvant chemotherapy or palliative chemotherapy (details are provided in **Supplementary Material** **Table S1**). For any precise tumor treatment method, including the PTC model, its predicted drug efficacy rate should be consistent with the patient’s clinical response rate of this drug among patients. In our PTC model, the predicted efficacy rate was determined by the cutoff value of the cell viability and the drug’s Ec. Therefore, after fixing the 0.7 cutoff value, we determined the Ec of a drug as the concentration such that the effective rate in the PTC assay was closest to the overall response rate of this drug in clinical practice which was assessed in 272 breast cancer patients with neoadjuvant chemotherapy or advanced treatment (**Table S2**). We used the PTC samples of 12 patients as the training cohorts to determine the Ec values of nine drugs. The Ec of all drugs used in this study was determined. Three replications were performed for each drug sample pair (**Figure S1**).

Because some drugs are dependent on exposure time while others are not, the drug exposure time is uniformly set to 24 h to ensure adequate and uniform exposure time.

## Case description

### Case 1

**Figure 1:**
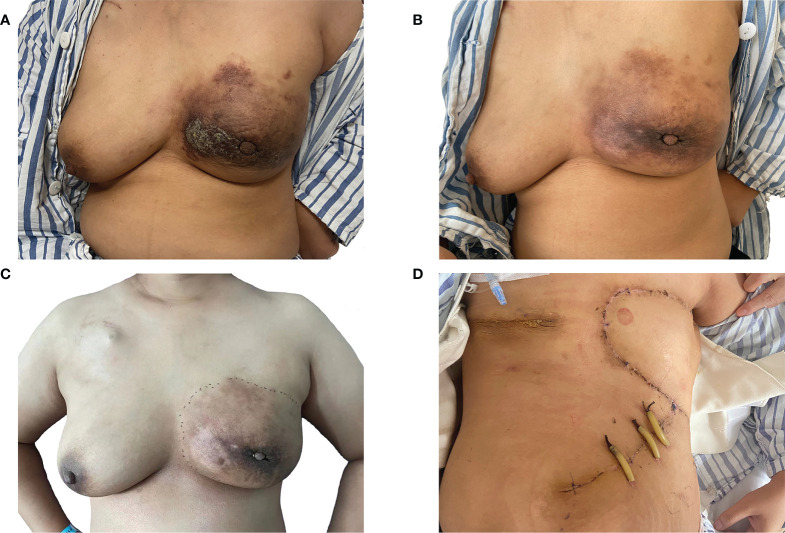
Evaluation of the therapeutic effect after chemotherapy. **(A)** A frontal view of the patient first presenting with a red and swollen left-side breast; **(B)** a frontal view of the redness and swelling of the patient’s left breast decreasing obviously after six cycles of chemotherapy; **(C)** a frontal view of the patient completing 10 cycles of chemotherapy prior to a planned mastectomy; **(D)** a frontal view of the patient after bilateral mastectomy and local rotation skin flap grafting.

**Figure 2:**
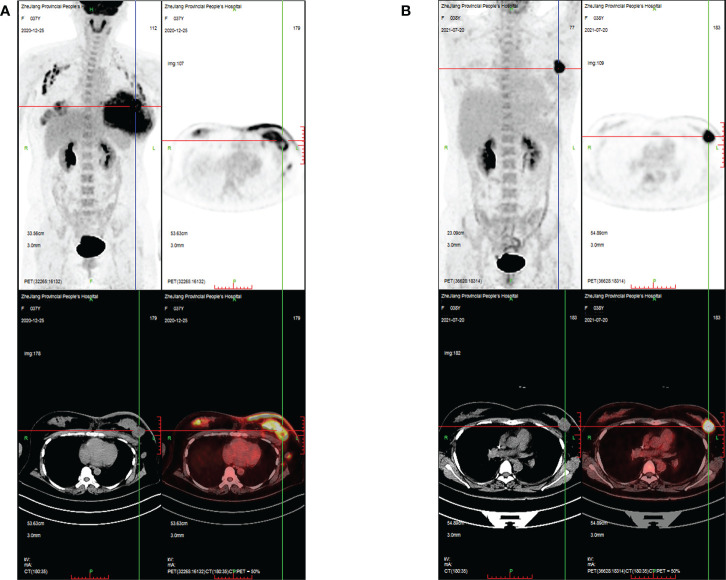
PET/CT images evaluating the changes in breast masses and metastatic lymph nodes during treatment in case 1. **(A)** PET/CT images showing intense FDG uptake in the bilateral breast, bilateral axilla, left clavicular area, and left upper mediastinum at the time of initial diagnosis; **(B)** PET/CT images showing that areas of high FDG uptake in the breast and lymph nodes were significantly reduced after 10 cycles of chemotherapy.

**Figure 3:**
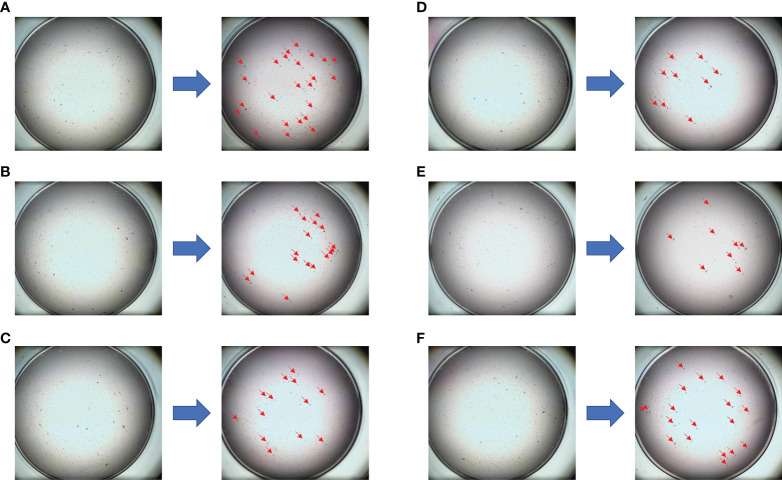
The drug sensitivity results of the PTC model for individualized treatment in case 1. **(A)** Comparison of trastuzumab before and after dosing; **(B)** comparison of epirubicin + cyclophosphamide before and after dosing; **(C)** comparison of vinorelbine + capecitabine before and after dosing; **(D)** comparison of albumin paclitaxel + capecitabine before and after dosing; **(E)** comparison of albumin paclitaxel + carboplatin before and after dosing; **(F)** negative control (NC) group.

A 37-year-old Chinese woman who was in lactation presented with a red and swollen left-side breast. Examination revealed 20 * 15 cm redness and swelling in the left breast and 4 * 3 cm mass in the upper quadrant of the right breast (**Figure 1A**). Core needle biopsies were performed on bilateral breast masses and revealed invasive ductal carcinoma [immunohistochemistry: left—estrogen receptor (ER) (−), progesterone receptor (PR) (−), human epidermal growth factor receptor 2 (HER2) (1+), Ki67 (+35%); right—ER (−), PR (−), HER2 (1+), Ki67 (+30%)]. Furthermore, positron emission tomography/computed tomography (PET/CT) showed multiple lymph node metastases in the bilateral axilla, left clavicular area, and left upper mediastinum, and her final TNM stage was T4N3M1 (**Figure 2A**). Individualized treatment was screened with the use of the PTC model, and the drug sensitivity results are included in **Figure 3** and **Figure S2** (details are available in **Supplementary Material** **Table S3**). Finally, compared with the other treatments, the albumin paclitaxel (125 mg/m^2^) plus carboplatin (AUC = 2) d1, 8, 1/21d regimen showed a higher killing rate of the tumor cells (47%) and was selected. After six cycles of chemotherapy, the tumor size was reduced obviously (**Figure 1B**), but the drop in platelet count was also significant. Thus, the regimen was adjusted to albumin paclitaxel (260 mg/m^2^, d1, 1/21d) plus capecitabine (150 mg BID, d1–14, 1/21d) for maintenance therapy based on previous drug sensitivity results with the PTC model. After 10 cycles of chemotherapy in total, PET/CT was performed again and indicated a significant reduction of tumor and lymph nodes (**Figure 2B**). Finally, to improve her quality of life, bilateral mastectomy and local rotation skin flap grafting were performed (**Figures 1C, D**). The final pathological results indicated a pathologic complete response (pCR) of the right side, with Miller–Payne (MP) grade 5, and MP grade 3 of the left side, with a residual tumor size of 2.5 * 2 cm. By now, the progression-free survival (PFS) has reached 20 months.

### Case 2

**Figure 4:**
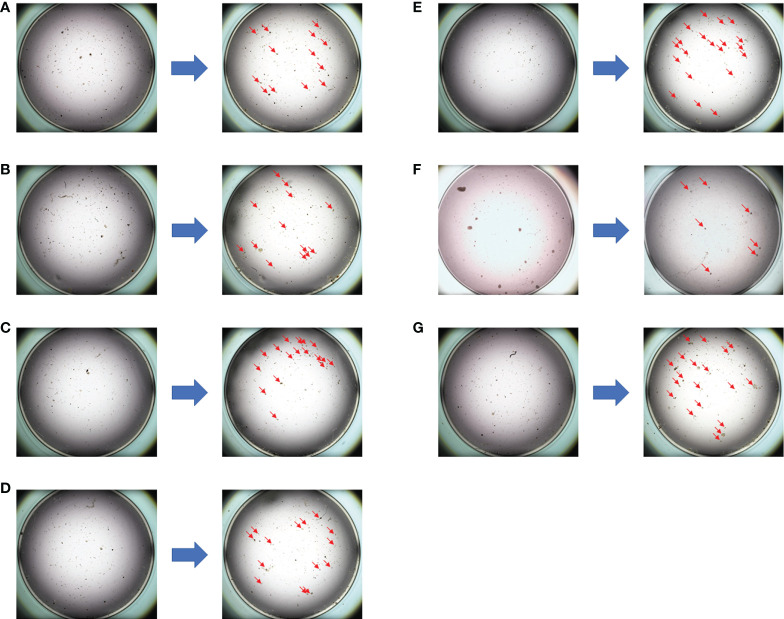
The drug sensitivity results of the PTC model for individualized treatment in case 2. **(A)** Comparison of albumin paclitaxel + capecitabine + trastuzumab before and after dosing; **(B)** comparison of albumin paclitaxel + carboplatin + trastuzumab before and after dosing; **(C)** comparison of albumin paclitaxel + carboplatin before and after dosing; **(D)** comparison of pyrotinib + capecitabine before and after dosing; **(E)** comparison of epirubicin before and after dosing; **(F)** comparison of albumin paclitaxel + trastuzumab + pertuzumab before and after dosing; **(G)** negative control (NC) group.

A 50-year-old Chinese woman presented with a 3 * 2-cm right breast mass. Core needle biopsy indicated invasive ductal carcinoma [immunohistochemistry: ER (−), PR (−), HER2 (3+), Ki67 (+80%)]. In addition, PET/CT showed multiple bone metastases and right axillary lymph node metastasis, and the final stage was T2N1M1. The PTC model was used for drug sensitivity screening (**Figure 4** and **Figure S3**; details are available in **Supplementary Material** **Table S4**). The PTH (albumin paclitaxel 260 mg/m^2^ d1, trastuzumab 8 mg/kg d1 followed by 6 mg/kg d1, and pertuzumab 840 mg d1 followed by 420 mg d1, 1/21d) regimen showed a better tumor cell killing rate of 70% and was finally selected. In addition, zoledronic acid was used to inhibit bone metastasis. After four cycles of chemotherapy, the tumor size was reduced obviously. Finally, right mastectomy and axillary lymph node dissection were performed, and the pathological results indicated a pCR of the tumor with MP grade 5. By now, the PFS has reached 18 months.

### Case 3

A 37-year-old Chinese woman presented with a 2.5 * 1.5-cm left breast mass. Core needle biopsy indicated invasive ductal carcinoma [immunohistochemistry: ER (−), PR (−), HER2 (2+), Ki67 (+30%)]. Fluorescence *in-situ* hybridization (FISH) was performed and the result was negative. Thus, the EC * 4 (pharmorubicin 90 mg/m^2^ plus cyclophosphamide 600 mg/m^2^ d1, 1/21d)–T * 4 (docetaxel 100 mg/m^2^ d1, 1/21d) regimen was used for neoadjuvant chemotherapy. After eight cycles of neoadjuvant chemotherapy, modified radical mastectomy was performed, and the pathological results revealed invasive ductal carcinoma [MP grade 3, immunohistochemistry: ER (−), PR (−), HER2 (2+), Ki67 (+20%)]. Then, FISH was performed again andthe result was still negative. However, 2 months later, thepatient was confirmed with chest wall metastases [immunohistochemistry: ER (−), PR (−), HER2 (2+), Ki67 (+10%)]. The PTC model was used for drug sensitivity screening (**Figure S4**), and the results indicated that anti-HER2 therapy was effective. Then, FISH was performed the third time, and the result turned out to be positive. Finally, the PTH (albumin paclitaxel 260 mg/m^2^ d1, trastuzumab 8 mg/kg d1 followed by 6 mg/kg d1, and pertuzumab 840 mg d1 followed by 420 mg d1, 1/21d) regimen was selected, and the chest wall metastases disappeared completely. By now, the PFS has reached 12 months.

### Case 4

A 56-year-old woman presented with a 3 * 2-cm right breastmass. A preoperative puncture was performed and showed invasive ductal carcinoma of the right breast (immunohistochemistry: ER (−), PR (−), HER2 (3+), Ki67 (+35%)] and negative axillary lymph node. All organs (including the lungs) showed no signs of metastasis during preoperative evaluation. Therefore, modified radical mastectomy and sentinel lymph node biopsy were performed, and the postoperative pathological report revealed invasive ductal carcinoma of the right breast [2.5 * 2.0 cm, immunohistochemistry: ER (−), PR (−), HER2 (3+), Ki67 (+70%)] and negative sentinel lymph node (0/5). One month later, CT showed multiple pulmonary nodules that had not been detected on preoperative CT and were considered metastatic (**Figure S5A**). Her final TNM stage was T2N0M1. Thus, the PTH (docetaxel 100 mg/m^2^ d1, trastuzumab 8 mg/kg d1 followed by 6 mg/kg d1, and pertuzumab 840 mg d1 followed by 420 mg d1, 1/21d) regimen was selected according to the guidelines. However, pulmonary nodules gradually increased and became larger after four cycles of treatment (**Figure S5B**). PET/CT suggested multiple metastatic nodules in both lungs, the largest of which was approximately 9 * 7 mm (**Figure S6A**). Thus, we implemented a PTC drug sensitivity test to screen effective drugs for individualized treatment. All drugs selected for the PTC sensitivity test were selected according to the guidelines and the clinical experience of experienced physicians. Interestingly, the PTC sensitivity test results suggested that compared with chemotherapy combined with targeted therapy, the corresponding chemotherapy regimen alone had similar tumor cell lethality (**Figures S7**, **S8**). This finding also demonstrated that the treatment regimen previously used (the PTH regimen) was ineffective. In addition, the pulmonary nodules were punctured, and the histopathological diagnosis was consistent with lung metastasis from breast cancer [immunohistochemistry: ER (−), PR (−), HER2 (1+), Ki67 (+30%)]. This result suggested that the patient’s HER2 status had changed, being positive in the primary tumor and negative in the metastatic lung nodule. This was consistent with our test results and might explain why targeted therapy based on the PTC sensitivity test was ineffective. Finally, the NCb (vinorelbine 25 mg/m^2^, d1, 8, carboplatin AUC = 2, d1, 8, 1/21d) plus targeted therapy (trastuzumab 6 mg/kg d1, pertuzumab 420 mg d1, 1/21d) regimen was selected based on the PTC drug sensitivity screening results. After four cycles of treatment, the pulmonary nodules almost completely disappeared (**Figures S5C**, **S6B**). At present, the patient has achieved a PFS time of 14 months.

## Discussion

The leading causes of death in patients with advanced breast cancer are tumor metastasis and drug resistance (3, 7). Increasing evidence has indicated that patient-derived tumor models could present human tumor biology and evaluate the potential clinical responses (8–10). Although patient-derived tumor xenografts (PDXs) were reported to be a precise measurement of drug screening, it was difficult to generate sufficient organoids for drug screening within 2 to 3 weeks from small tissue samples (6, 9, 11). To address the defects of previous technologies, the PTC model was emphasized, which could be a method of long-term maintenance and expansion of primary tumor cells in a Matrigel-free condition (6, 12), and it was reported as a structural and functional unit which could recapitulate the original tumors according to genotype, phenotype, and drug response within 2 weeks (6). Furthermore, a previous study has demonstrated that the PTC model in breast cancer can express ER, PR, and HER2 status similar to those of the original tumor (6). To ensure the accuracy and stability of drug sensitivity, specific culture conditions and an accurate cutoff value of the PTC model were established (6, 13). The consistency of PTC cell viability in different wells as well as the consistency between the predicted results of the PTC model and the patient’s clinical response has been demonstrated (6). Furthermore, the PTC model was proved to be a tool for personalized treatment selection which had a prediction accuracy of 93% (6, 14).

Our case report first evaluated the application value of the PTC model in advanced breast cancer and filled in its lack of clinical validation in the individualized treatment of breast cancer. In these four cases of individualized treatment for advanced breast cancer, the PTC model showed a good predictive effect. The tumor size was reduced significantly or even disappeared completely through clinical, radiological, or pathological evaluation with the help of the PTC model for selecting an individualized therapy regimen. Patients who responded to the treatments were reported to have a better OS; thus, the PTC model, which was reported to increase the pCR rate, might have a certain effect on improving the OS (15, 16). Furthermore, the drug sensitivity test results of the PTC model were consistent with pathological molecular typing and the actual clinical drug resistance of the patients. For example, the drug sensitivity results of the PTC model showed that anti-HER2 therapy was insensitive to the tumor in case 1, which was pathologically confirmed as triple negative breast cancer, while in case 3, the drug sensitivity results of the PTC model indicated that anti-HER2 therapy was effective to the patient whose first two FISH tests were negative. Finally, it was confirmed that her HER2 status was positive. Moreover, in case 4, the primary tumor was HER2 positive, but the PTC drug sensitivity test results suggested that lung metastases were insensitive to anti-HER2 therapy. The histopathological findings from the biopsy of pulmonary nodules in case 4 finally confirmed that the patient’s HER2 status had changed from positive in the primary tumor to negative in the lung metastasis, which was consistent with the PTC drug sensitivity test results. In addition, the results of the PTC sensitivity test confirmed that the previously used therapeutic regimen was ineffective, suggesting that it had high value in predicting the drug resistance of tumors. Therefore, the PTC model might be used for evaluating the efficacy of chemotherapy and targeted therapy and for estimating pathological molecular typing, and it demonstrated good value in guiding individualized treatment.

In summary, our case report first evaluated the application value of the PTC model in advanced breast cancer, and the PTC model might be used as an efficient tool for drug resistance screening and for selecting a better personalized treatment, although further study is needed to prove the validity and stability of the PTC model in drug screening.

## Data availability statement

The original contributions presented in the study are included in the article/**Supplementary Material**. Further inquiries can be directed to the corresponding author.

## Ethics statement

The studies involving human participants were reviewed and approved by Ethics Committee of Zhejiang Provincial People’s Hospital. The patients/participants provided their written informed consent to participate in this study. Written informed consent was obtained from the individual(s) for the publication of any potentially identifiable images or data included in this article.

## Author contributions

WJ and WZ conceived the study, collected and analyzed the data, and co-wrote the first draft. HJ and SF evaluated the histopathological findings, and edited the manuscript. JX and JW were directly involved in the treatment of the patient. All authors contributed to the article and approved the submitted version.

## Funding

This study was supported by the Public Welfare Technology Application Research Project of Zhejiang Province under Grant No. LGF21H160030, Zhejiang Provincial Natural Science Foundation of China together with Zhejiang Society for Mathematical Medicine (No. LSY19F020002), and Medical and Health Science and Technology Project of Zhejiang Province (2021KY061 and 2023KY046).

## Conflict of interest

The authors declare that the research was conducted in the absence of any commercial or financial relationships that could be construed as a potential conflict of interest.

## Publisher’s note

All claims expressed in this article are solely those of the authors and do not necessarily represent those of their affiliated organizations, or those of the publisher, the editors and the reviewers. Any product that may be evaluated in this article, or claim that may be made by its manufacturer, is not guaranteed or endorsed by the publisher.

## Supplementary material

Supplementary Figure 1 Drug efficacy concentration (Ec). The determination of Ec was based upon the efficacy rate (ER) closest to the overall response rate (OR).

Supplementary Figure 2 The summary of fold changes of tumor cells before and after drug dosing in case 1. It indicating albumin paclitaxel+carboplatin regimen showed the highest tumor cells killing rate of 47% in the PTCs model, and anti-HER2 therapy was insensitive to triple negative breast cancer.

Supplementary Figure 3 The summary of fold changes of tumor cells before and after drug dosing in case 2. Albumin paclitaxel+ trastuzumab+ pertuzumab regimen showing the highest tumor cells killing rate of 70% in the PTCs model.

Supplementary Figure 4 The summary of fold changes of tumor cells before and after drug dosing in case 3. It indicating anti-HER2 therapy was effective of PTCs model in case 3.

Supplementary Figure 5 Axial CT lung window demonstrating changes in pulmonary nodules during treatment in Case 4. **(A)** Axial CT lung window demonstrating multiple pulmonary nodules, which was considered pulmonary metastasis. **(B)** Axial CT lung window demonstrating pulmonary nodules were markedly enlarged after four cycles of PTH (docetaxel+trastuzumab+pertuzumab) treatment. **(C)** Axial CT lung window demonstrating that pulmonary nodules almost disappeared after four cycles of NCbPH (vinorelbine+carboplatin+trastuzumab+pertuzumab) treatment, which was based on CTC drug sensitivity test results.

Supplementary Figure 6 PET/CT images evaluating changes in pulmonary nodules during treatment in Case 4. **(A)** PET CT image showing intense FDG uptake in bilateral pulmonary nodules after four cycles of PTH treatment. **(B)** PET CT image showing that the area of high FDG uptake in the lung was almost completely eliminated after four cycles of NCbPH treatment, which was based on CTC drug sensitivity test results.

Supplementary Figure 7 PTCs drug sensitivity test results for individualized treatment in Case 4. **(A)** Comparison of trastuzumab before and after dosing; **(B)** Comparison of PTH(docetaxel+trastuzumab+pertuzumab) before and after dosing; **(C)** Comparison of albumin paclitaxel+PH (albumin paclitaxel+trastuzumab+pertuzumab) before and after dosing; **(D)** Comparison of NCb(vinorelbine+carboplatin) before and after dosing; **(E)** Comparison of NCbPH (vinorelbine +carboplatin +trastuzumab +pertuzumab) before and after dosing; **(F)** negative control(NC) group. The results indicating that tumor cells were insensitive to anti-HER-2 therapy and PTH regimen, and this was consistent with pathological molecular typing and previous clinical practice in which PTH regimen showed poor efficacy. NCbPH and NCb regimen showing a higher killing rate of tumor cells(61.7% and 60.3%).

Supplementary Figure 8 Summary of the fold changes in PTCs before and after drug dosing in Case 4.

Supplementary Table 1 Detailed patient information for evaluating the clinical efficacy.

Supplementary Table 2 Summary of efficacy and concentrations of drugs used in this study.

Supplementary Table 3 The detailed data of changes in the number of tumor cells before and after drug dosing in case 1.

Supplementary Table 4 The detailed data of changes in the number of tumor cells before and after drug dosing in case 2.

## References

[1] Siegel RL Miller KD Fuchs HE Jemal A. Cancer statistics, 2021. CA Cancer J Clin (2021) 71(1):7–33. doi: 10.3322/caac.21654 33433946

[2] Allemani C Matsuda T Di Carlo V Harewood R Matz M Niksic M. Global surveillance of trends in cancer survival 2000-14 (CONCORD-3): analysis of individual records for 37 513 025 patients diagnosed with one of 18 cancers from 322 population-based registries in 71 countries. Lancet. (2018) 391(10125):1023–75. doi: 10.1016/S0140-6736(17)33326-3 PMC5879496 29395269

[3] Zhang W Xia W Lv Z Ni C Xin Y Yang L. Liquid biopsy for cancer: Circulating tumor cells, circulating free DNA or exosomes? Cell Physiol Biochem (2017) 41(2):755–68. doi: 10.1159/000458736 28214887

[4] Pan B Li X Zhao D Li N Wang K Li M. Optimizing individualized treatment strategy based on breast cancer organoid model. Clin Transl Med (2021) 11(4):e380. doi: 10.1002/ctm2.380 33931968 PMC8012563

[5] Harris EER. Precision medicine for breast cancer: The paths to truly individualized diagnosis and treatment. Int J Breast Canc (2018) 2018:4809183. doi: 10.1155/2018/4809183 PMC5971283 29862084

[6] Yin S Xi R Wu A Wang S Li Y Wang C. Patient-derived tumor-like cell clusters for drug testing in cancer therapy. Sci Transl Med (2020) 12(549):eaaz1723. doi: 10.1126/scitranslmed.aaz1723 32581131

[7] Hoadley KA Yau C Hinoue T Wolf DM Lazar AJ Drill E. Cell-of-Origin patterns dominate the molecular classification of 10,000 tumors from 33 types of cancer. Cell. (2018) 173(2):291–304 e6. doi: 10.1016/j.cell.2018.03.022 29625048 PMC5957518

[8] Gao H Korn JM Ferretti S Monahan JE Wang Y Singh M. High-throughput screening using patient-derived tumor xenografts to predict clinical trial drug response. Nat Med (2015) 21(11):1318–25. doi: 10.1038/nm.3954 26479923

[9] Stewart E Federico SM Chen X Shelat AA Bradley C Gordon B. Orthotopic patient-derived xenografts of paediatric solid tumours. Nature. (2017) 549(7670):96–100. doi: 10.1038/nature23647 28854174 PMC5659286

[10] Byrne AT Alferez DG Amant F Annibali D Arribas J Biankin AV. Interrogating open issues in cancer precision medicine with patient-derived xenografts. Nat Rev Canc (2017) 17(4):254–68. doi: 10.1038/nrc.2016.140 28104906

[11] Rossi G Manfrin A Lutolf MP. Progress and potential in organoid research. Nat Rev Genet (2018) 19(11):671–87. doi: 10.1038/s41576-018-0051-9 30228295

[12] Ding RB Chen P Rajendran BK Lyu X Wang H Bao J. Molecular landscape and subtype-specific therapeutic response of nasopharyngeal carcinoma revealed by integrative pharmacogenomics. Nat Commun (2021) 12(1):3046. doi: 10.1038/s41467-021-23379-3 34031426 PMC8144567

[13] Eisenhauer EA Therasse P Bogaerts J Schwartz LH Sargent D Ford R. New response evaluation criteria in solid tumours: revised RECIST guideline (version 1. 1). Eur J Cancer (2009) 45(2):228–47. doi: 10.1016/j.ejca.2008.10.026 19097774

[14] Jiang S Zhao H Zhang W Wang J Liu Y Cao Y. An automated organoid platform with inter-organoid homogeneity and inter-patient heterogeneity. Cell Rep Med (2020) 1(9):100161. doi: 10.1016/j.xcrm.2020.100161 33377132 PMC7762778

[15] Cortazar P Zhang L Untch M Mehta K Costantino JP Wolmark N. Pathological complete response and long-term clinical benefit in breast cancer: the CTNeoBC pooled analysis. Lancet (2014) 384(9938):164–72. doi: 10.1016/S0140-6736(13)62422-8 24529560

[16] Kuerer HM Newman LA Smith TL Ames FC Hunt KK Dhingra K. Clinical course of breast cancer patients with complete pathologic primary tumor and axillary lymph node response to doxorubicin-based neoadjuvant chemotherapy. J Clin Oncol (1999) 17(2):460–9. doi: 10.1200/JCO.1999.17.2.460 10080586

