# Supplementary figures and images for: Case report: Individualized treatment of advanced breast cancer with the use of the patient-derived tumor-like cell cluster model
Source: Front Oncol. 2022 Oct 31;12:897984. doi: 10.3389/fonc.2022.897984 (PMC9659609; doi:10.3389/fonc.2022.897984)

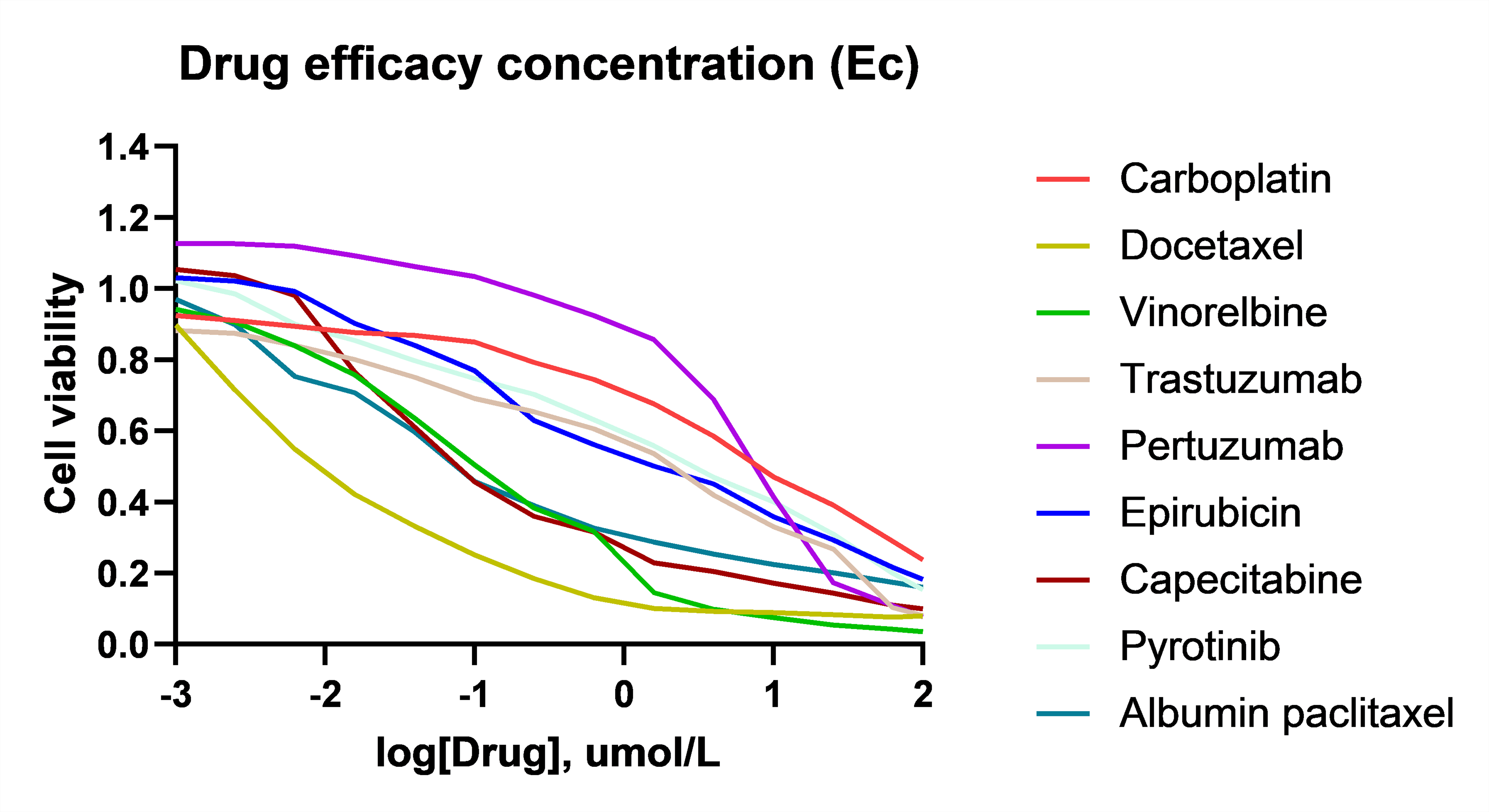

Supplement: Supplementary Figure 1 — Drug efficacy concentration (Ec). The determination of Ec was based upon the efficacy rate (ER) closest to the overall response rate (OR). [file Image_1.tif]

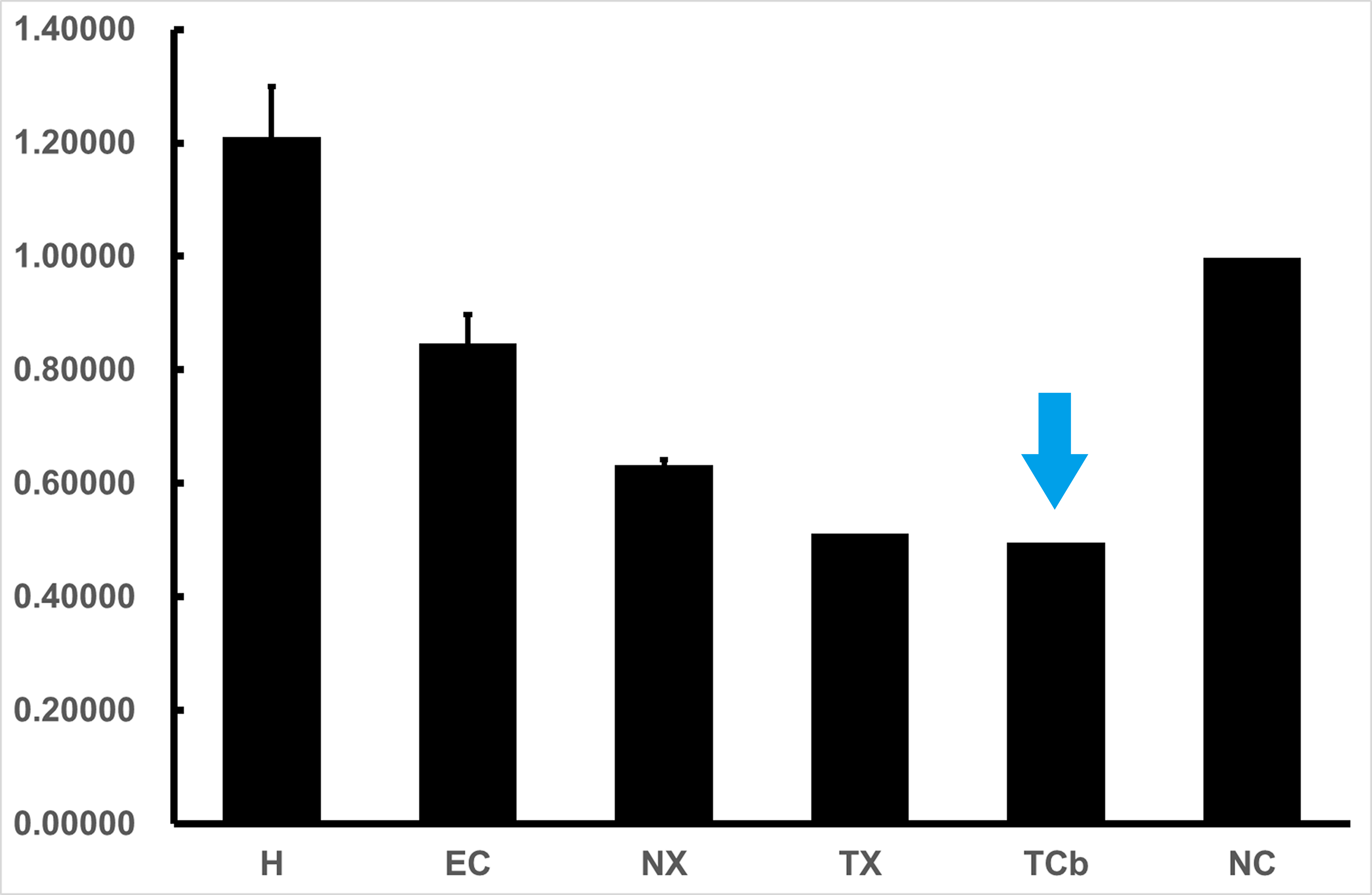

Supplement: Supplementary Figure 2 — The summary of fold changes of tumor cells before and after drug dosing in case 1. It indicating albumin paclitaxel+carboplatin regimen showed the highest tumor cells killing rate of 47% in the PTCs model, and anti-HER2 therapy was insensitive to triple negative breast cancer. [file Image_2.tif]

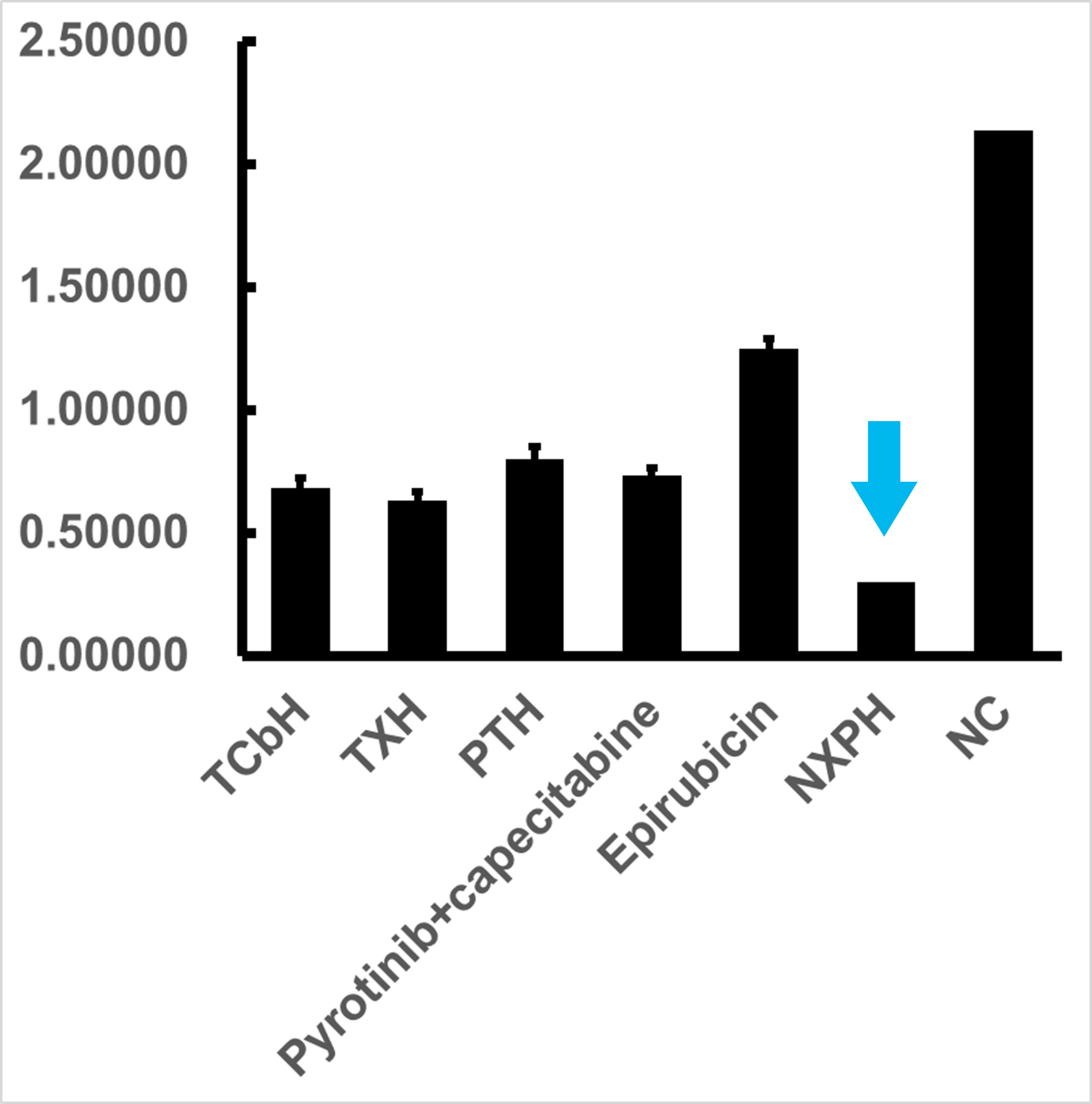

Supplement: Supplementary Figure 3 — The summary of fold changes of tumor cells before and after drug dosing in case 2. Albumin paclitaxel+ trastuzumab+ pertuzumab regimen showing the highest tumor cells killing rate of 70% in the PTCs model. [file Image_3.tif]

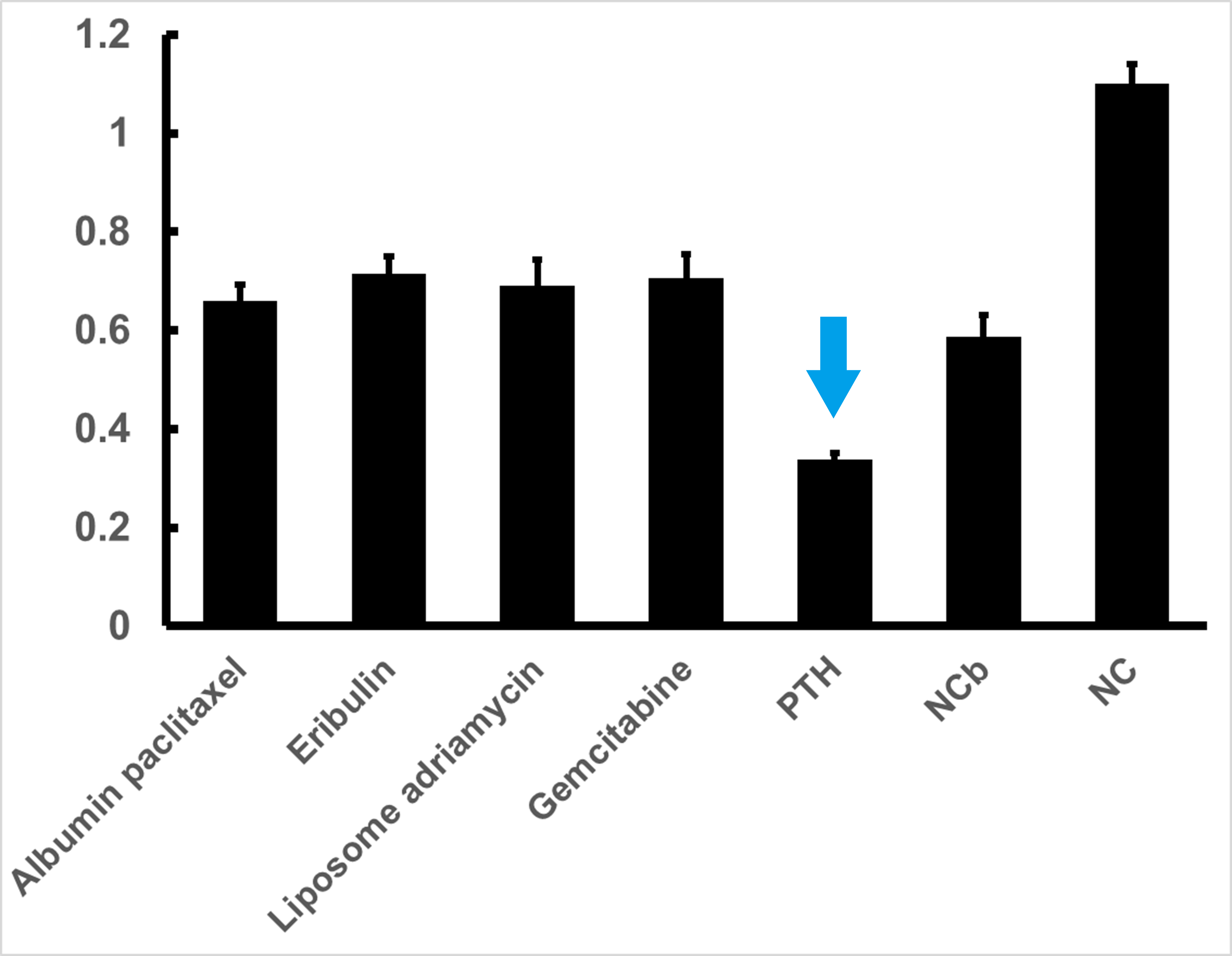

Supplement: Supplementary Figure 4 — The summary of fold changes of tumor cells before and after drug dosing in case 3. It indicating anti-HER2 therapy was effective of PTCs model in case 3. [file Image_4.tif]

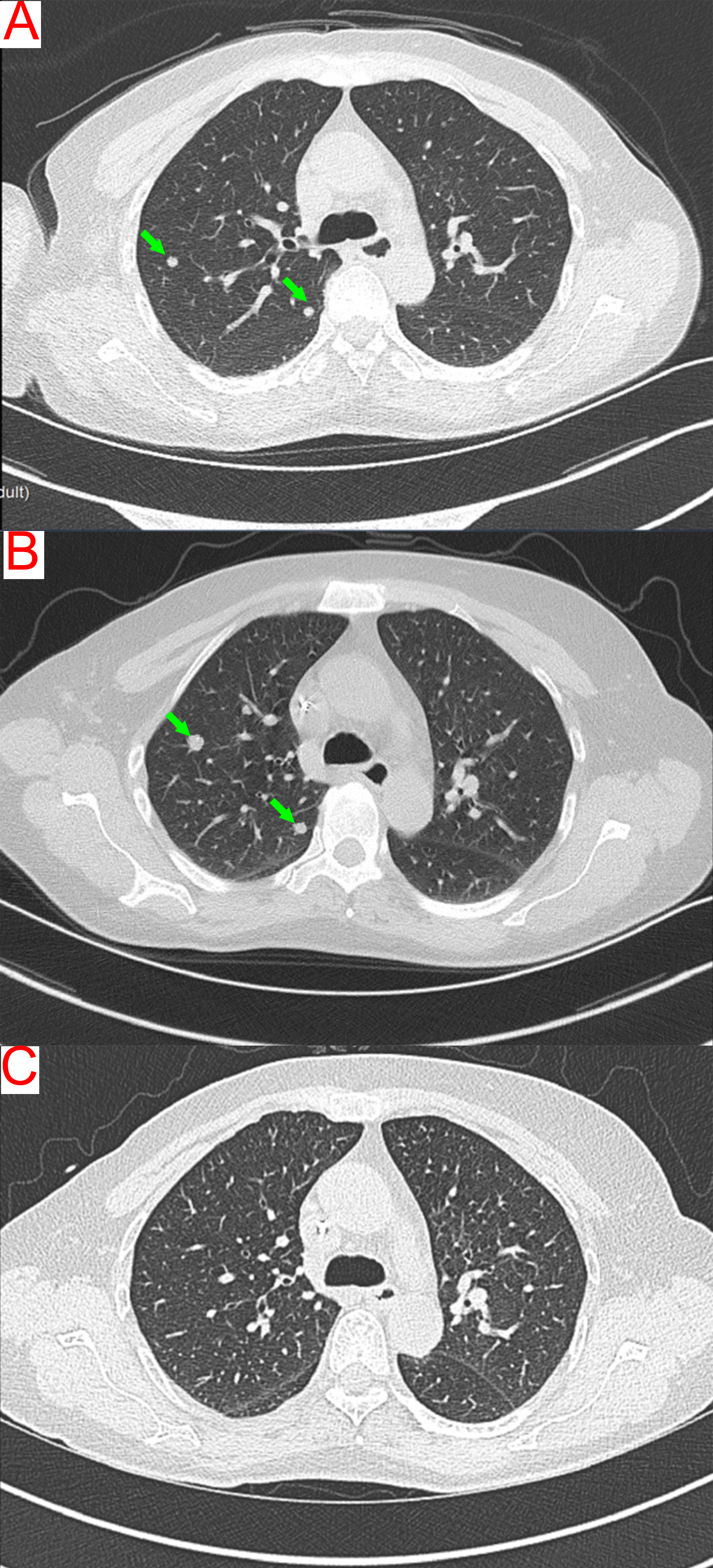

Supplement: Supplementary Figure 5 — Axial CT lung window demonstrating changes in pulmonary nodules during treatment in Case 4. (A) Axial CT lung window demonstrating multiple pulmonary nodules, which was considered pulmonary metastasis. (B) Axial CT lung window demonstrating pulmonary nodules were markedly enlarged after four cycles of PTH (docetaxel+trastuzumab+pertuzumab) treatment. (C) Axial CT lung window demonstrating that pulmonary nodules almost disappeared after four cycles of NCbPH (vinorelbine+carboplatin+trastuzumab+pertuzumab) treatment, which was based on CTC drug sensitivity test results. [file Image_5.tif]

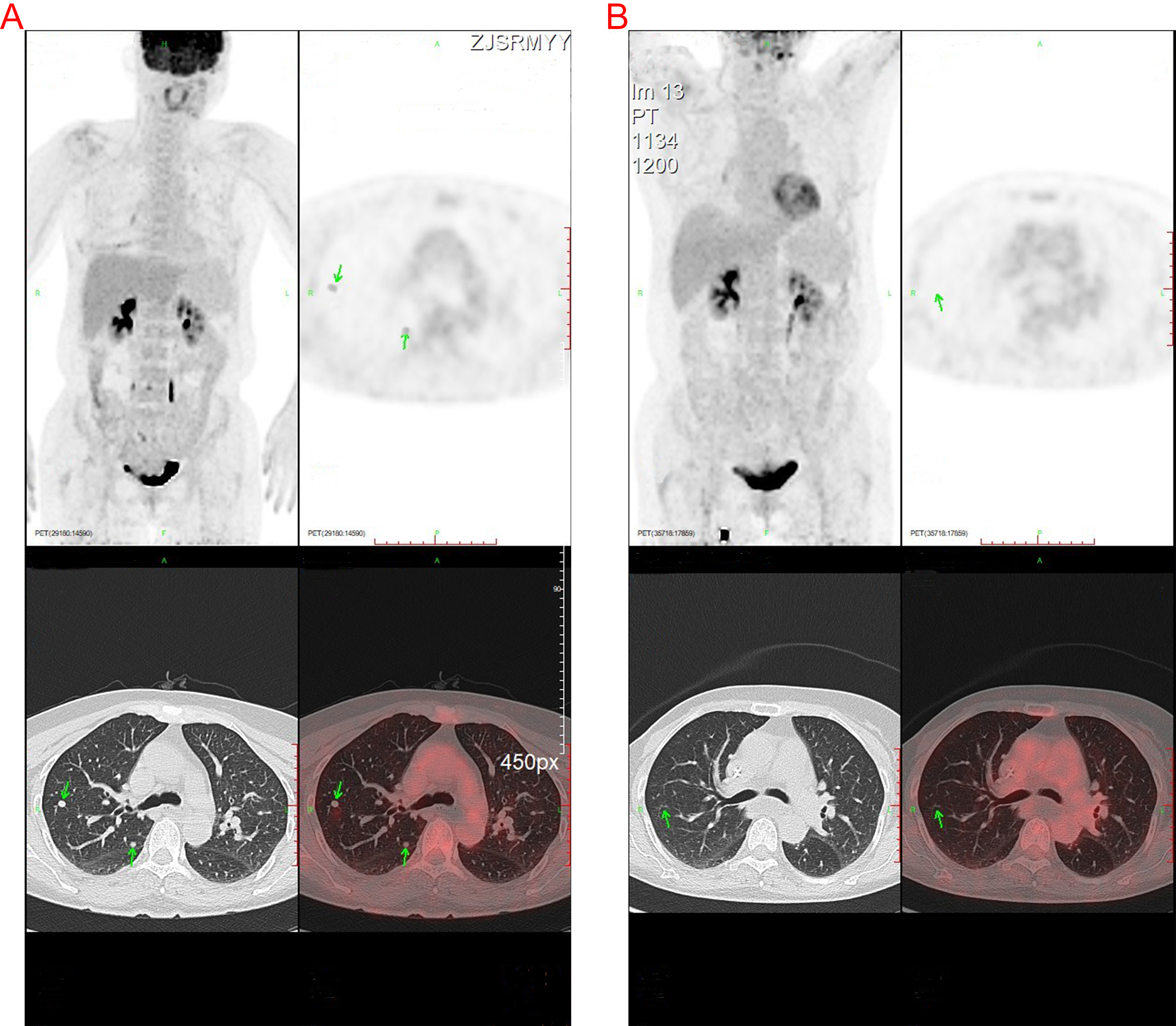

Supplement: Supplementary Figure 6 — PET/CT images evaluating changes in pulmonary nodules during treatment in Case 4. (A) PET CT image showing intense FDG uptake in bilateral pulmonary nodules after four cycles of PTH treatment. (B) PET CT image showing that the area of high FDG uptake in the lung was almost completely eliminated after four cycles of NCbPH treatment, which was based on CTC drug sensitivity test results. [file Image_6.tif]

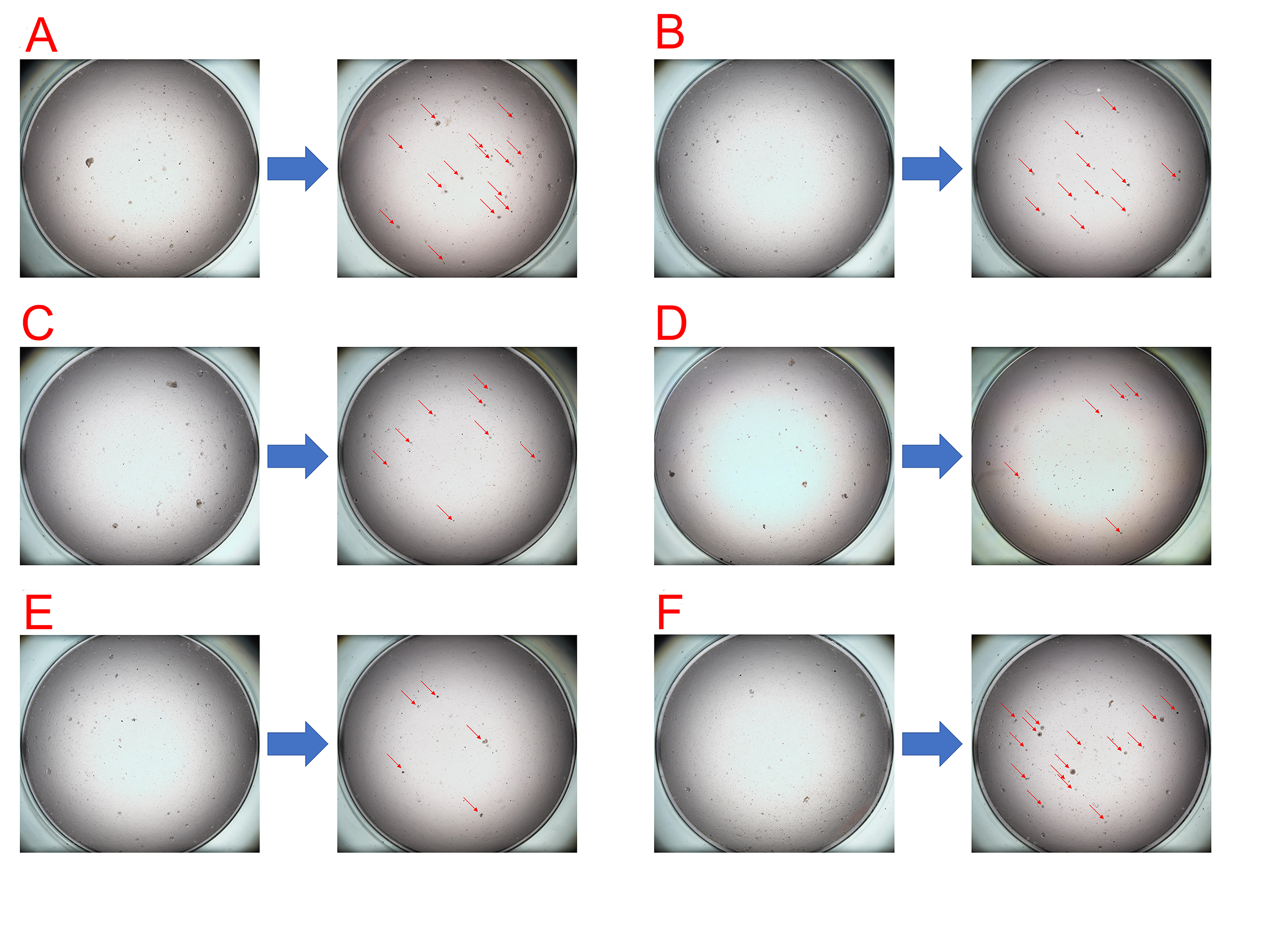

Supplement: Supplementary Figure 7 — PTCs drug sensitivity test results for individualized treatment in Case 4. (A) Comparison of trastuzumab before and after dosing; (B) Comparison of PTH(docetaxel+trastuzumab+pertuzumab) before and after dosing; (C) Comparison of albumin paclitaxel+PH (albumin paclitaxel+trastuzumab+pertuzumab) before and after dosing; (D) Comparison of NCb(vinorelbine+carboplatin) before and after dosing; (E) Comparison of NCbPH (vinorelbine +carboplatin +trastuzumab +pertuzumab) before and after dosing; (F) negative control(NC) group. The results indicating that tumor cells were insensitive to anti-HER-2 therapy and PTH regimen, and this was consistent with pathological molecular typing and previous clinical practice in which PTH regimen showed poor efficacy. NCbPH and NCb regimen showing a higher killing rate of tumor cells(61.7% and 60.3%). [file Image_7.tif]

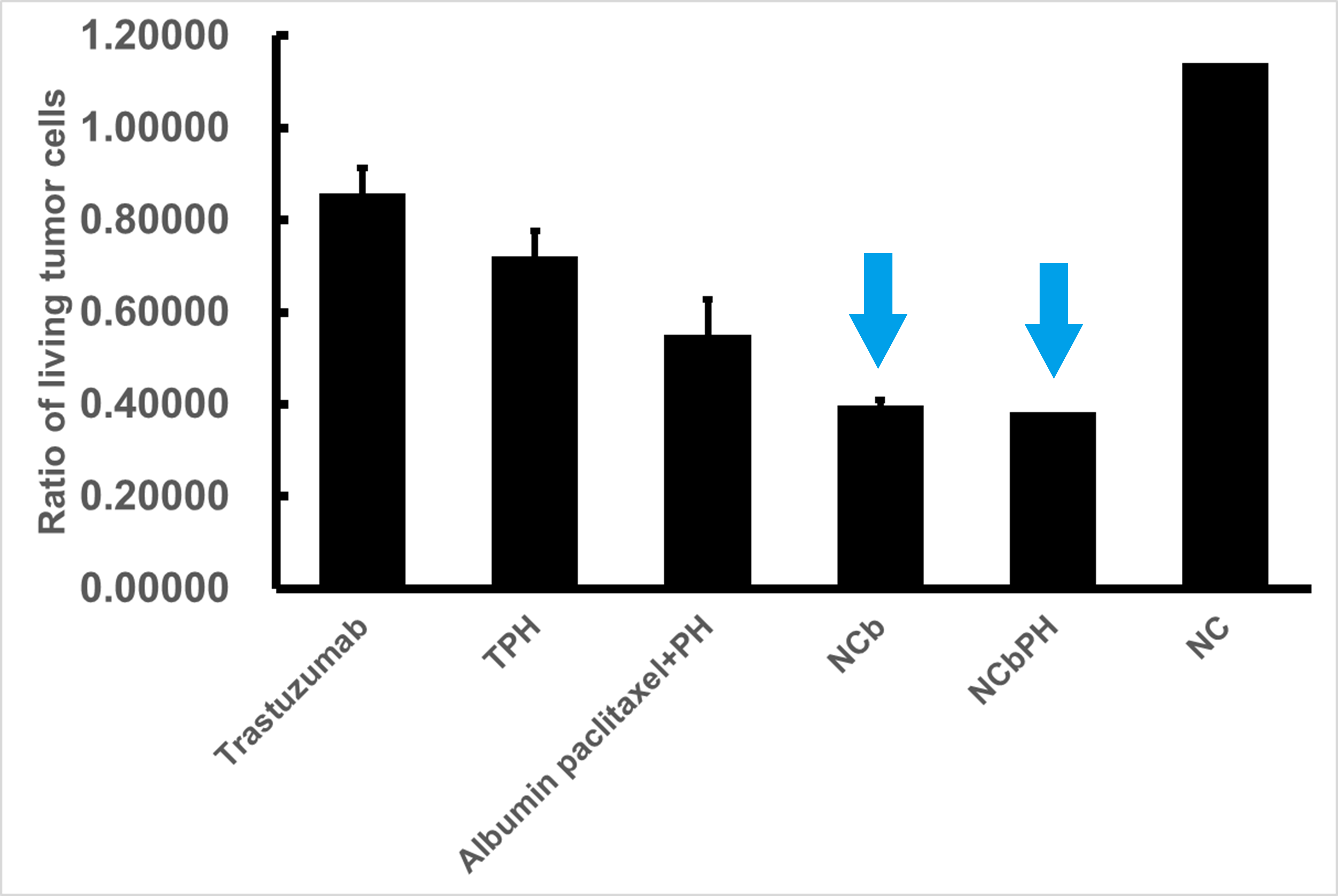

Supplement: Supplementary Figure 8 — Summary of the fold changes in PTCs before and after drug dosing in Case 4. [file Image_8.tif]
