# Supplementary material for: Case report: Individualized treatment of advanced breast cancer with the use of the patient-derived tumor-like cell cluster model
Source: Front Oncol. 2022 Oct 31;12:897984. doi: 10.3389/fonc.2022.897984 (PMC9659609; doi:10.3389/fonc.2022.897984)
Supplement: Supplementary Table 2 — Summary of efficacy and concentrations of drugs used in this study. [file Table_2.docx]

**Table S2** **Summary of efficacy and concentrations of drugs used in this study.**

| **Regimens** | **Overall response rate (ORR)** | **Efficacy rate(ER)** | **Efficacy concentration [μmol/L]** |
| --- | --- | --- | --- |
| Docetaxel | 190/250(76%) | 75% | 0.1 |
| Vinorelbine | 4/8(50%) | 50% | 0.1 |
| Capecitabine | 11/20(55%) | 54% | 0.1 |
| Carboplatin | 18/27(66.7%) | 65% | 30 |
| Albumin paclitaxel | 12/21(57.1%) | 55% | 0.1 |
| Pyrotinib | 2/5(40%) | 40% | 1 |
| Epirubicin | 2/5(40%) | 39% | 0.3 |
| Trastuzumab | 74/98(75.5%) | 75% | 30 |
| Pertuzumab | 28/32(87.5%) | 86% | 30 |
